# Checklist of gypsophilous vascular flora in Italy

**DOI:** 10.3897/phytokeys.103.25690

**Published:** 2018-07-18

**Authors:** Carmelo Maria Musarella, Antonio Jesús Mendoza-Fernández, Juan Francisco Mota Alessandro Alessandrini3, Gianluigi Bacchetta Salvatore Brullo6, Orazio Caldarella Giampiero Ciaschetti8, Fabio Conti Luciano Di Martino8, Amedeo Falci Lorenzo Gianguzzi11, Riccardo Guarino Aurelio Manzi13, Pietro Minissale Sergio Montanari14, Salvatore Pasta Lorenzo Peruzzi16, Lina Podda Saverio Sciandrello17, Leonardo Scuderi, Angelo Troia, Giovanni Spampinato

**Affiliations:** 1 Department of AGRARIA, Mediterranean University of Reggio Calabria, Loc. Feo di Vito, 89122 Reggio Calabria, Italy; 2 Department of Biology and Geology, CECOUAL, University of Almeria, Desp. 1.101. Edificio Cientifico Tecnico II – B. Ctra. Sacramento s/n. La Cañada de San Urbano, 04120 Almeria, Spain; 3 Regional Institute for Cultural Heritage. Via Galliera 21 40121 Bologna Italy; 4 Centre for the Conservation of Biodiversity (CCB), Life and Environmental Sciences Department, University of Cagliari,Viale S. Ignazio da Laconi 11-13, 09123 – Cagliari, Italy; 5 Hortus Botanicus Karalitanus (HBK), University of Cagliari, Viale S. Ignazio da Laconi 9-11, 09123 Cagliari, Italy; 6 Department of Biological, Geological and Environmental Sciences, University of Catania, Via A. Longo 19, 95125 Catania, Italy; 7 Via Maria SS. Mediatrice n° 38, 90129 Palermo, Italy; 8 Majella National Park, Via Badia 28 I-67039 Sulmona (AQ), Italy; 9 Center of floristic research of the Apennine – School of Biosciences and Veterinary Medicine, University of Camerino, Italy; 10 Via Libertà n° 200, 93100 Caltanissetta, Italy; 11 Department of Agricultural and Forest Sciences. Università degli Studi di Palermo, Italy; 12 Department STEBICEF, Botanical Unit. University of Palermo, Italy; 13 Via Peligna n° 228, 66010 Gessopalena (CH), Italy; 14 SSNR (Società per gli Studi Naturalistici della Romagna), C.P. 143, 48012 Bagnacavallo (RA), Italy; 15 Department of Biology, University of Fribourg, Chemin du Musée 10, CH-1700, Fribourg, Switzerland; 16 Department of Biology, Unit of Botany, University of Pisa, Via Derna 1, 56126, Pisa, Italy; 17 Centre for the Conservation and Management of Nature and Agroecosystems (CUTGANA). University of Catania, Italy; 18 Via Andromaca n° 60, 91100 Trapani, Italy

**Keywords:** Edaphism, Gypsophyte, Habitats Directive, Plant preservation

## Abstract

Our understanding of the richness and uniqueness of the flora growing on gypsum substrates in Italy has grown significantly since the 19^th^ century and, even today, new plant species are still being discovered. However, the plants and plant communities, growing on gypsum substrates in Italy, are still a relatively unknown subject.

The main aim of this paper was to elaborate a checklist of the Italian gypsophilous flora, to increase knowledge about this peculiar flora and for which conservation efforts need to be addressed.

Through a structured group communication process of experts (application of the Delphi technique), a remarkable number of experienced Italian botanists have joined together to select focal plant species linked to gypsum substrates. From the results obtained, 31 plant species behave as absolute or preferent taxa (gypsophytes and gypsoclines) and form the ‘core’ Italian gypsophilous flora. The most abundant life forms were chamaephytes and hemicryptophytes, belonging to Poaceae and Brassicaceae; as for chorotypes, the most represented are Mediterranean and narrow endemics. By improving on previously available information about the flora with a clear preference for gypsum in Italy, this undertaking represents an important contribution to the knowledge of a habitat which is today considered a priority for conservation.

## Introduction

The relationship between local bedrock types and vegetation cover has long been highlighted. Andrea Cesalpino, in *De plantis libri XVI* (1583), had already documented the existence of endemic plant species on the Italian serpentines. The term ‘edaphism’ – interpreted as a ‘geobotanical phenomenon giving rise to particular floras on certain substrates’ (Font Quer 1977) or ‘those physical and chemical effects induced on living beings by the soil’ (Sarmiento 2001) – has been used extensively in Europe since the 19^th^ century (Parsons 1976, Kruckeberg 2002). The species and the plant assemblages growing on gypsum provide a clear example of the strict relationship between soil and vegetation, as many plant species grow exclusively or preferentially on such peculiar substrates. This geobotanical pattern occurs in more than 70 countries worldwide (Pérez-García et al. 2017). Nevertheless, the gypsicolous substrates represent a largely underrated or ignored habitat, with serious consequences for both flora and fauna conservation. These habitats host sparse and scattered vegetation, since the gypsum outcrops often represent geological islands interrupting the uniformity of other surrounding landscapes. Local evolutionary processes have probably been facilitated by the geographical isolation of outcrops, so that the appearance of several plant species with a narrow distribution have been favoured (Moore et al. 2014). This kind of geographic speciation could have been complemented by some selective pressure that might have favoured the survival of certain plant lineages on nutrient-unbalanced, water-limited soils which are unfavourable to the establishment of most plants (Merlo et al. 1998, 2001, Bolukbasi et al. 2016).

There is an abundance of accurate information concerning gypsophilous plant communities in Spain (Mota et al. 2011), but not for those other European countries where such substrata also occur. In Cyprus, for instance, it is only the outcrop located at Rizoelia National Park that are known, but unfortunately it seems that both local flora and vegetation are degraded due to several impact factors (EIONET 2008). The first scholar to study the gypsophilous flora of Italy was Macchiati (1888). There have been some studies in mainland Italy and Sicily which have increased our knowledge about this flora, e.g. Gallo (2014). Such previous studies on both the rate of endemism and risk of extinction amongst these plant communities pointed out that ‘Mediterranean gypsum vegetation’ should be considered a Priority Habitat (*1520) for conservation according to the 92/43 EU Directive (Anonymous 1992). Since no specific national law has yet been enacted in Italy (Fenu et al. 2017, Rossi et al. 2016), this directive theoretically represents the major instrument for plant conservation. Several Italian gypsum outcrops have been identified as SCI (Sites of Community Importance) and SCZ (Special Conservation Zones) e.g. Piano di Gestione Complessi Gessosi M. Conca, SIC ITA050006 (IUCN UNEP-WCMC 2014, Mento 2008). However, information about gypsum vegetation in Italy is still deficient: in fact, neither its current area, nor the recent trends in term of quantity and quality (last 50 years) are known (Loidi 2016). An accurate checklist of Italian gypsophilous flora has not yet been produced; hence, only published investigations concerning these flora and vegetation could be used as a reference guide. When taking into account that conservation policies at any level must be based on scientific assessments about habitats, species conservation status and existing threats (IUCN 2001; Joppa et al. 2013), the exhaustive knowledge about the flora typical of gypsum outcrops in Italy would be a crucial step towards the application of any sort of conservation measure (Mota et al. 2004, 2011, Martínez-Hernández et al. 2011, 2015).

Therefore, the aims of this research were (i) to elaborate a checklist of Italian gypsophilous vascular flora through a structured group communication process of experts; (ii) to expand the knowledge of this flora type to which conservation efforts need to be addressed; (iii) to examine the spectrum of taxonomical groups, life forms and chorotypes of this flora. Through this approach, the comparison between the gypsophilous flora of Italy and that of other countries was carried out in order to detect common phylogenetic, functional and biogeographic patterns that allow a better understanding of the gypsophily phenomenon at European and global levels.

## Methods

Several approaches have been proposed to elucidate which plant species can be considered as best linked to gypsum substrates (Mota et al. 2016). However, coping with an extremely species-rich flora over a wide territory such as Italy, using the Delphi technique (Hasson et al. 2000) resulted in being the most effective way to build a checklist of gypsophilous flora by using the so-called ‘expert criterion’ (Mota et al. 2008, 2009).

### The Delphi technique

The Delphi technique is a structured, anonymous and iterative survey undertaken by a panel of ‘experts’, which enables a group of individuals to collectively address a complex problem through a structured group communication process. This method has been applied in ecology to fill in data gaps (Eycott et al. 2011), through the experience of the participants (Ochoa-Gaona et al. 2010). The technique can be classified into four categories relevant to ecology and conservation (Hasson and Keeney 2011). One of these categories is Decision Delphi, which is used to identify focal species for conservation.

Our scheme comprised two rounds of semi-structured questionnaires, each followed by an aggregation of responses and anonymous feedback from the experts. The number of rounds was limited and adapted according to the time available. An increased number of rounds would make the process more time-consuming.

### Preparation of the first round of the questionnaire

A semi-structured survey, drawing from evidence based on published literature, was designed. The initial listing of taxa included species issuing from bibliographical references, which recorded the presence of these taxa on gypsum substrates (See Appendix 1: Methodology References for detailed information).

### Selection and invitation of a panel of experts

**Figure 1.**
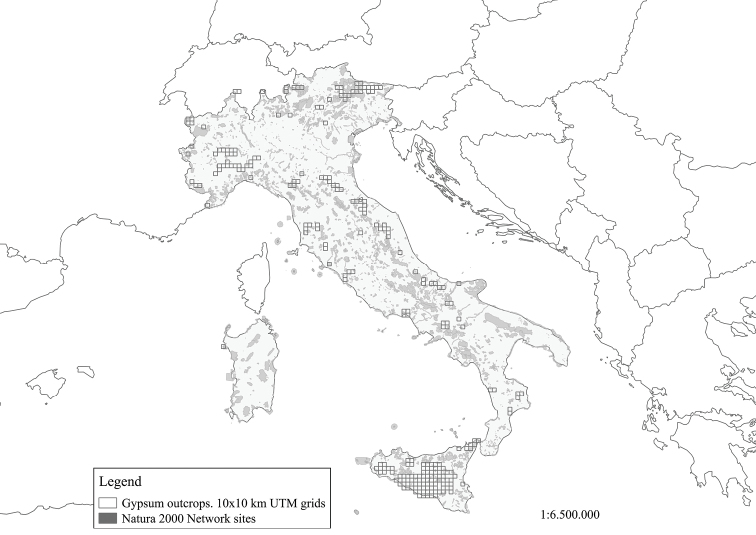
Italian gypsum outcrop presences in 10×10 km UTM grids.

Participants from a great diversity of backgrounds were included (e.g. teachers, scientists, conservationists, non-governmental organisations, policy-makers, environmental managers and technicians) in order to obtain a wide range of perspectives and minimise bias arising from self-interest or information preferences. The participants included the co-authors of this article, i.e. experts situated in the Italian peninsula and Sardinia – hereinafter Italy (9) – and in Sicily (11). The reason why the number of Sicilian botanists involved is greater than those from mainland Italy is due to the considerable extension and importance that gypsum outcrops have in Sicily (Fig. 1).

### Collection and analysis of the completed questionnaire for the first round

Once the preliminary plant catalogue was elaborated, the complete list was submitted to the group of experts (Musarella et al. 2016). The experts were clearly asked to base their gypsophily assessment for each plant species only on their personal field experience in order to avoid any judgement based on bibliographical references or other sources of information. In addition, the peers were provided with a series of hierarchical criteria according to the Likert (1932) scale, a method where participants were asked to rank their responses on a scale of ‘one to five’ (Table 1) where ‘one’ indicated an ‘absolute absence on gypsum outcrops (plants that avoid gypsum or, at most, may eventually occur on this substrate)’, while ‘five’ indicated ‘absolute preference (the considered plant species only grows on gypsum)’.

**Table 1.** Likert scale ranking for the gypsophilous character of the taxa.

**5**	Strictly gypsophile species; that is, species that do not live outside gypsum substrates (except accidentally). **ALWAYS GROW ON GYPSUM**	**Strict gypsophyte**
**4**	Species with great preference for gypsum and which are found very rarely outside this substrate. **ALMOST ALWAYS GROW ON GYPSUM**	**Preferential gypsophyte**
**3**	Species that live on gypsum, but which can also live on other substrates. If they live on many other different types of soil, they will not fit into this category. For example, if they live on limestone, marls and gypsum they could fall into this category. At least, it is as abundant (or almost) on gypsum as it is on other types of substrates. **GYPSUM AND OTHER HIGHLY RELATED SUBSTRATES ARE THEIR PREFERRED HABITATS**	**Subgypsophyte**
**2**	Species that may be abundant on gypsum, although they could be even more frequent on other types of substrates. **CLEARLY MORE COMMON OUTSIDE OF GYPSUM**	**Gypsovag**
**1**	Very rare species on gypsum or absent on this type of soil. **NEVER (OR ONLY ACCIDENTALLY) ON GYPSUM**	**Accidental**

### Preparation and analysis of second round questionnaire

The collated responses from the first round were used to prepare a second questionnaire. The experts were requested to add new taxa candidates to be subsequently evaluated by the panel (Spampinato et al. 2016). The second round questionnaire was administered only to respondents who participated to the first round. The responses were collated and analysed using quantitative measures. Statistical summaries were generated for the responses, central tendencies (mean, median) and the levels of dispersion (standard deviation and interquartile ranges) of each taxon. The results were compiled into a report, which was used in the next step as feedback and described quantitative details to the participants.

### Plant species data

Information about plant species included in the checklist was collected: (i) taxonomic rank (Bartolucci et al. 2018); (ii) family (Peruzzi 2010); (iii) chorology and (iv) life form (Pignatti 1982); (v) conservation status (Rossi et al. 2014); and (vi) functional groups (narrow gypsophiles, wide gypsophiles or gypsovags) according to Bolukbasi et al. (2016) and Palacio et al. (2007). See Suppl. material 1 for complete information.

### Checklist data analysis

Considering that the plant species assessment was made with a quantitative but discontinuous scale, median values could be useful criteria for selecting the gypsophilous species (Mota et al. 2011). According to the proposed Likert scale, species like gypsoclines (subgypsophytes or preferential gypsophytes) with median values 3 and 4 could be considered; and those with median values >4, such as gypsophytes.

Several statistical t-student and ANOVA tests were performed (SPSS ver. 22.0.0.0. IBM SPSS Statistics). Only taxa with gypsophily median values ≥2 were assessed in order to exclude ‘casual occurring taxa’. This analysis examined statistical differences between gypsum affinity (gypsophily), functional groups (narrow gypsophile, wide gypsophile and gypsovag) and distribution (Endemic, Mediterranean s.l., European, Eurasiatic/Widespread).

## Results

### Decision Delphi technique

The first round of the questionnaire comprised 115 plant taxa. However, experts included more than 69% of other taxa growing on Italian gypsum substrates. This fact implied that, during the second round questionnaire, the panel of experts made assessments of 380 taxa (Suppl. material 1). All these taxa were assessed by at least one of the experts. The set of 380 taxa received on average 8.95 valuations out of 20 (i.e. almost half of the specialists, 44.60%, gave an assessment). The average number of evaluations from the mainland Italian botanists was 2.29, compared to 6.65 from the Sicilian botanists.

The species in this catalogue belong to 59 different families. As far as the taxonomic spectrum of the 380 taxa is concerned, the most represented families were Asteraceae (14%), Poaceae (9.5%), Fabaceae (8.4%), Lamiaceae (6.3%) and Orchidaceae (6.1%). Moreover, the percentages of life forms on this preliminary list were as follows: therophytes (28.7%), hemicryptophytes (24%), geophytes (17.1%), chamaephytes (16.3%), nanophanerophytes (7.9%) and phanerophytes (6.1%). According to their distribution, two groups were clearly highlighted. The first one is composed of species with Mediterranean distribution (49.5%); the second included Italian endemic species (16.8%). The rest of the species were grouped (in smaller percentages) under Eurimediterranean, Submediterranean, European and Euroasiatic/Widespread species. Considering the conservation status of the species in the preliminary list, only seven taxa had IUCN extinction risk assessments and five of them were considered to be threatened according to IUCN categories: CR [*Aizoanthemopsis hispanicum* (L.) Klak., *Limonium calcarae* (Tod. ex Janka) Pignatti and *Astragalus raphaelis* Ferro]; EN [*Allosorus persicus* (Bory) Christenh.]; VU [*Tripolium sorrentinoi* (Tod.) Raimondo & Greuter].

### Checklist of Italian gypsophilous flora

The consensus, established amongst the responses of the panel of experts, produced the first checklist of Italian gypsophilous flora. A tiny group of 31 species out of the 380 preliminary taxa (8.16%) obtained median values over 3 from the experts’ assessments, so that they can be considered as gypsophiles or gypsoclines (Table 2).

**Table 2.** Checklist of Italian gypsophilous flora. Species are listed in decreasing order of Median. Life-form: Therophyte (T), Chamaephyte (Ch), Hemicryptophyte (H), Nanophanerophyte (NP), Geophyte (G). Distribution: Endemic (Endem.), Mediterranean (Medit.), Sub-Mediterranean (S-Medit.), European (Europ.), Widespread (Wide.). IUCN category: Endangered (EN), Least Concern (LC) species. Number of assessment (NA). Median (Median) and average (Mean) values of experts’ assessments. Standard deviation (SD). Median values from mainland Italy and Sicilian experts separately (Mainland Med/Sicily Med).

Species	Synonym	Family	Life form	Chorology	IUCN	NA	Score	Median	Mean	SD	Mainland Median	Sicily Median
*Chaenorhinum rupestre* (Guss.) Speta	*Chaenorhinum exile* (Coss. & Kralik) Lange	Plantaginaceae	T	S-Medit.	-	11	55	5.00	5.00	0.00	-	5.00
*Festuca gypsophila* Hack.	*Ctenopsis gypsophila* (Hack.) Paunero)	Poaceae	T	Medit.	-	1	5	5.00	5.00	-	-	5.00
Sedum gypsicola Boiss. & Reuter subsp. trinacriae Afferni		Crassulaceae	Ch	Medit.	-	11	51	5.00	4.64	0.67	-	5.00
Petrosedum ochroleucum (Chaix) Niederle subsp. mediterraneum (L.Gallo) Nieder*le*		Crassulaceae	Ch	Endem.	-	9	38	5.00	4.22	1.30	-	5.00
*Allosorus persicus* (Bory) Christenh.	*Cheilanthes persica* (Bory) Mett. ex Kuhn, *Notholaena persica* Bory	Pteridaceae	H	Medit.	EN	5	21	5.00	4.20	1.10	5.00	3.00
*Artemisia pedemontana* Balb.		Asteraceae	Ch	Europ.	-	2	9	4.50	4.50	0.71	5.00	4.00
Stipa austroitalica Martinovský subsp. frentana Moraldo & Ricceri		Poaceae	H	Endem.	LC	5	21	4.00	4.20	0.84	4.50	4.00
Diplotaxis harra (Forssk.) Boiss. subsp. crassifolia (Raf.) Maire	*Diplotaxis crassifolia* (Raf.) DC.	Brassicaceae	Ch	S-Medit.	-	11	45	4.00	4.09	0.94	-	4.00
Brassica villosa Biv. subsp. tineoi (Lojac.) Raimondo & Mazzola		Brassicaceae	Ch	Endem.	-	11	43	4.00	3.91	1.30	-	4.00
*Erysimum metlesicsii* Polatschek		Brassicaceae	H	Endem.	-	11	40	4.00	3.64	0.92	-	4.00
*Limonium catanzaroi* Brullo		Plumbaginaceae	H	Endem.	-	5	18	4.00	3.60	1.67	-	4.00
*Limonium optimae* Raimondo		Plumbaginaceae	H	Endem.	-	5	18	4.00	3.60	1.67	-	4.00
*Reaumuria vermiculata* L.		Tamaricaceae	NP	S-Medit.	-	4	13	4.00	3.25	1.50	-	4.00
Gypsophila arrostii Guss. subsp. arrostii		Caryophyllaceae	Ch	Endem.	-	11	36	3.00	3.27	0.90	-	3.00
Matthiola fruticulosa (L.) Maire subsp. coronopifolia (Sm.) Giardina & Raimondo		Brassicaceae	Ch	Endem.	-	1	3	3.00	3.00	-	-	3.00
*Allium moschatum* L.		Amaryllidaceae	G	Europ.	-	1	3	3.00	3.00	-	3.00	-
Elymus elongatus (Host) Runemark subsp. elongatus	*Elymus obtusiflorus* (DC.) Conert	Poaceae	G	Europ.	-	1	3	3.00	3.00	-	-	3.00
*Thapsia meoides* (Desf.) Guss.		Apiaceae	H	Medit.	-	4	11	3.00	2.75	0.50	-	3.00
Matthiola fruticulosa (L.) Maire subsp. fruticulosa	*Matthiola tristis* (L.) R.Br.	Brassicaceae	Ch	Europ.	-	11	30	3.00	2.73	0.90	2.00	3.00
*Visnaga crinita* (Guss.) Giardina & Raimondo	*Ammi crinitum* Guss.	Apiaceae	T	Endem.	-	5	13	3.00	2.60	0.89	-	3.00
Stipa barbata Desf. subsp. barbata		Poaceae	H	Medit.	-	7	18	3.00	2.57	0.79	-	3.00
*Linum decumbens* Desf.		Linaceae	T	Medit.	-	9	23	3.00	2.56	0.53	-	3.00
Phagnalon rupestre (L.) DC. subsp. illyricum (H.Lindb.) Ginzb.		Asteraceae	Ch	Medit.	-	9	23	3.00	2.56	0.88	3.00	2.50
Astragalus caprinus L. subsp. huetii (Bunge) Podlech		Fabaceae	H	Endem.	-	11	28	3.00	2.55	1.13	-	3.00
*Capparis sicula* Veill.	Capparis spinosa L. subsp. spinosa var. canescens Coss.	Capparaceae	NP	Medit.	-	11	28	3.00	2.55	0.82	1.00	3.00
*Teucrium luteum* (Mill.) Degen	Teucrium polium L. subsp. aureum (Schreb.) Arcang.	Lamiaceae	Ch	Medit.	-	7	16	3.00	2.29	0.95	-	3.00
*Lygeum spartum* L.		Poaceae	H	S-Medit.	-	9	20	3.00	2.22	0.97	3.00	2.50
*Cachrys sicula* L.	*Hippomarathrum siculum* (L.) Hoffm. & Link	Apiaceae	H	Medit.	-	5	11	3.00	2.20	1.10	-	3.00
*Parapholis strigosa* (Dumort.) C.E.Hubb.		Poaceae	T	Medit.	-	5	11	3.00	2.20	1.10	1.00	3.00
*Suaeda vera* J.F.Gmel.	*Suaeda fruticosa* (L.) Forssk. (auct. Fl. Ital.)	Amaranthaceae	NP	Wide.	-	5	11	3.00	2.20	1.10	1.00	3.00
*Parapholis incurva* (L.) C.E.Hubb.	*Lepturus incurvus* (L.) Druce	Poaceae	T	Medit.	-	7	15	3.00	2.14	1.07	1.00	3.00

The 31 Italian gypsophilous taxa, on average, received 11 evaluations by experts (i.e. more than 55% of specialists evaluated this group): a result which increased the average number of assessments that the 380 taxa considered as the preliminary list received by more than 10%. Specifically, only the 9 species group composed of *Chaenorhinum rupestre* (Guss.) Speta, Sedum gypsicola Boiss. & Reut. subsp. trinacriae Afferni, Brassica villosa Biv. subsp. tineoi (Lojac.) Raimondo & Mazzola, Diplotaxis harra (Forssk.) Boiss. subsp. crassifolia (Rafin.) DC., *Erysimum metlesicsii* Polatschek, Astragalus caprinus L. subsp. huetii (Bunge) Podlech, *Capparis sicula* Veill., Gypsophila arrostii Guss. subsp. arrostii and Matthiola fruticulosa (L.) Maire subsp. fruticulosa, was evaluated by 11 or more specialists.

Within the evaluation of the Italian Checklist of gypsophilous flora, 8 taxa obtained arithmetic-mean values higher than 4, whilst 8 taxa values were greater than or equal to 3. In the case of the median calculation, 5 species showed values equal to 5, 8 taxa reached values equal to or greater than 4 and 18 were equal to or above the median value 3.

**Figure 2.**
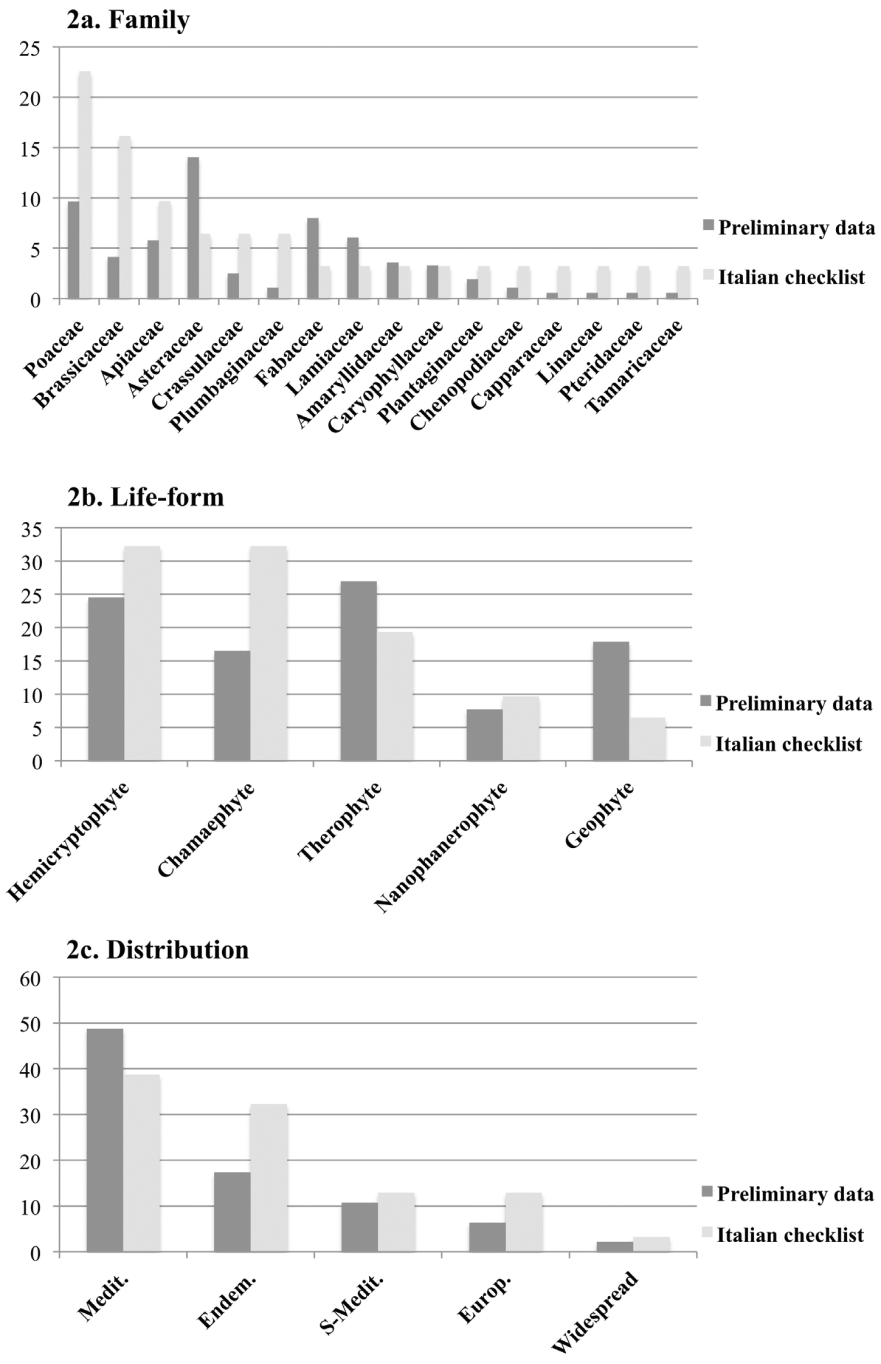
Percentage of taxa grouping by taxonomic families, life-forms and distribution and comparison between gypsophilous flora and preliminary data.

The plant species on the Italian Checklist are present in 16 families (Figure 2 and Table 3), amongst which the most abundant are Poaceae (22.6%), Brassicaceae (16.1%), Apiaceae (9.7%), Asteraceae, Crassulaceae and Plumbaginaceae (6.5%). Interestingly, these 6 families alone represented almost 70% of the total gypsophilous species. As regards life forms, the spectrum is as follows: chamaephytes (32.26%), hemicryptophytes (32.26%), therophytes (19.35%), nanophanerophytes (9.67%) and geophytes (6.45%). In terms of the distribution analysis, the Italian Checklist highlights exactly the same two groups of the initial list, with those species with Mediterranean distribution predominating (38.7%), followed by the group composed of Italian endemic species. In the case of this last group, the species percentage was double that in the same analysis on the preliminary list (32.3%) (Figure 2).

**Table 3.** Percentage of gypsophile taxa grouping by taxonomic families and a comparison between Italian and Spanish Checklists (Mota et al. 2011).

Family	Italian Checklist	Spanish Checklist
Amaryllidaceae	3.23	1.41
Apiaceae	9.68	1.41
Asteraceae	6.45	14.08
Brassicaceae	16.13	12.68
Campanulaceae	–	1.41
Capparaceae	3.23	–
Caryophyllaceae	3.23	8.45
Amaranthaceae	3.23	–
Cistaceae	–	4.23
Crassulaceae	6.45	1.41
Euphorbiaceae	–	1.41
Fabaceae	3.23	9.86
Frankeniaceae	–	1.41
Gentianaceae	–	1.41
Lamiaceae	3.23	11.27
Linaceae	3.23	–
Orobanchaceae	–	1.41
Plantaginaceae	3.23	5.63
Plumbaginaceae	6.45	12.68
Poaceae	22.58	4.23
Primulaceae	–	1.41
Pteridaceae	3.23	–
Resedaceae	–	4.23
Tamaricaceae	3.23	–

When the evaluations of the Sicilian experts are compared with those of experts from the peninsula (Table 2), the latter consider 23 species with gypsophily values higher than 3, although only 6 of them were included in the final checklist (19.4%). These species are *Artemisia pedemontana* Balb., *Allosorus persicus* (Bory) Christenh., Stipa austroitalica Martinovský subsp. frentana Moraldo & Ricceri, *Allium moschatum* L., *Lygeum spartum* L. and Phagnalon rupestre (L.) DC. subsp. illyricum (H.Lindb.) Ginzb. The Sicilian experts considered 32 taxa with gypsophily values equal to or greater than 3 and 28 of these species are present in the final checklist (90.3%).

Finally, in the case of Sardinia, where few gypsum outcrops are located in the northwest and in southeast of the island, local experts have highlighted the presence of three taxa (Euphorbia pithyusa L. subsp. pithyusa, Helichrysum italicum (Roth) G.Don subsp. tyrrhenicum (Bacch., Brullo & Giusso) Herrando, J.M.Blanco, L.Sáez & Galbany and Teucrium marum L. subsp. occidentale Mus, Mayol & Rossellò) which reached gypsophily values between 1.86 and 2.33 in the experts’ assessments.

### Statistical analysis

Statistical tests (t-student) showed significant differences between the groups of Italian endemic plants versus species with a wider distribution; the Italian endemics obtained a higher average value of gypsophily (Table 4). However, when comparing the gypsophily values and groups of taxonomic family or life form, no significant difference was found.

In addition, when considering the clustering performed by distribution, the ANOVA analysis showed the existence of significant differences in gypsophily values between the Italian endemics and those showing both Mediterranean and European distribution. This is not so for species with a wider distribution range, such as Eurasian and widespread taxa. However, this result could be an artefact due to the small size of this sample, since its average gypsophily value is the one that most differed from the endemic species group (Table 5a).

Finally, according to the grouping variables narrow gypsophile, wide gypsophile and gypsovag, the species considered as narrow gypsophile, showed on average the highest gypsophily values and reached maximum values. Both species regarded as narrow gypsophile and wide gypsophile showed statistically significant results with higher gypsophily values than those considered as gypsovags. Nevertheless, there were no significant differences between the groups of narrow gypsophile and wide gypsophile, so that this separation was not supported (Table 5b).

For further information about the statistical analyses performed see Suppl. material 2.

**Table 4.** *t-student* analyses by gypsophily level, taxa grouping by endemic and non-endemic species. Number of species (N). Average (AV). Standard deviation (SD). Standard error (SE).

	N	AV	SD	SE	*p*-value	95% confidence interval	
						Min	Max
Endemism	46	2.4000	0.7731	0.1153	**0.0030**	2.1680	2.6320
Rest	293	2.1460	0.4744	0.0285		2.0900	2.2020
Total	339	2.1810	0.5321	0.0296		2.1230	2.2390

* p-value < 0.05

**Table 5.** ANOVA analysis by gypsophily level. Average (AV). Standard deviation (SD). 5a) Grouping by distribution: Italian endemic, Mediterranean, European and Wide distribution. 5b) Grouping by functional group: narrow gypsophile, wide gypsophile and gypsovag.

a) Chorotype		AV	SD	*p*-value	95% confidence interval	
					Min	Max
Endemic	Mediterranean	**0.2358**	0.0868	**0.0350**	0.0120	0.4600
	European	**0.2917**	0.1038	**0.0270**	0.0240	0.5600
	Eurasiat/Widespread	0.3412	0.1498	0.1060	-0.0460	0.7280
Mediterranean	Endemic	-**0.2358**	0.0868	**0.0350**	-0.4600	-0.0120
	European	0.0558	0.0774	0.8890	-0.1440	0.2560
	Eurasiat/Widespread	0.1054	0.1329	0.8580	-0.2380	0.4490
European	Endemic	-**0.2917**	0.1038	**0.0270**	-0.5600	-0.0240
	Mediterranean	-0.0558	0.0774	0.8890	-0.2560	0.1440
	Eurasiat/Widespread	0.0495	0.1446	0.9860	-0.3240	0.4230
Eurasiat/Widespread	Endemic	-0.3412	0.1498	0.1060	-0.7280	0.0460
	Mediterranean	-0.1054	0.1329	0.8580	-0.4490	0.2380
	European	-0.0495	0.1446	0.9860	-0.4230	0.3240
**b) Functional group**		**AV**	**SD**	***p*-value**	**95% confidence interval**	
					**Min**	**Max**
Narrow gypsophile	Wide gypsophile	0.1524	0.1014	0.2910	-0.0860	0.3910
	Gypsovag	**1.6692**	0.0848	**0.0000**	1.4690	1.8690
Wide gypsophile	Narrow gypsophile	-0.1524	0.1014	0.2910	-0.3910	0.0860
	Gypsovag	**1.5168**	0.0596	**0.0000**	1.3760	1.6570
Gypsovag	Narrow gypsophile	-**1.6692**	0.0848	**0.0000**	-1.8690	-1.4690
	Wide gypsophile	-**1.5168**	0.0596	**0.0000**	-1.6570	-1.3760

* p-value < 0.05

## Discussion

*Ad hoc* investigations on gypsophily have been performed in only 12 countries and only five of these studies approached a functional perspective (Mota et al. 2016). However, documented gypsophilous flora can be found in at least 75 countries (Pérez-García et al. 2017, 2018).

This work provides the first Checklist of Italian gypsophytes, including 31 taxa showing a great affinity for this substrate, 12 of which can be unequivocally considered as strictly gypsophytes. In addition, a number of further species often found on these substrates is detailed. As mentioned before, although the studies on gypsophilous flora in Italy date as far back as the 19^th^ century (Macchiati 1888), there is no knowledge of the existence of detailed ecological, functional or phylogeographic studies. After this first analysis of Italian flora, it would not be possible to discard a second evaluation based on the information presented in this work and any new information generated in the future (e.g. Montanari et al. 2016). A new revision should not only take into account the vascular flora, but also the plant assemblages associated with these outcrops. It must be noted that Italy presents a complex natural scenario, with a strong North-South environmental gradient conditioning the composition and dynamics of plant communities. In the case of vegetation associated with gypsum outcrops, this gradient is remarkable and it is impossible to overlook the far greater aridity of the southernmost regions, which exaggerates the gypsophily phenomenon (Merlo et al. 1998, 2001). This probably explains why the largest contingent of gypsophytes in the Italian territory is concentrated in Sicily.

This research reinforces the idea that, provided there is no definitive criterion for establishing whether a species is a gypsophyte or not, the inductive approach based on ‘expert criterion’ is not only plausible, but perhaps the only one possible to establish the groundwork for future research on gypsophily. To further complicate this scenario, the same species may have different affinity levels for gypsum substrates in isolated territories: e.g. Sedum gypsicola subsp. trinacriae shows gypsophily median value of 5 in Italy, but in Spain, the nominal subspecies (S. gypsicola subsp. gypsicola) reached a median value of 4 in a previous study (Mota et al. 2011).

Both the taxonomical and life form spectra concerning the 31 gypsophytes on the Italian Checklist are largely in agreement with the data recorded in other areas of the Mediterranean Basin for this type of substrates (EIONET 2008, Bolukbasi et al. 2016). As far as the most represented families are concerned, the taxonomic spectrum of the strictly gypsophilous flora in Italy is similar. Brassicaceae and Poaceae are amongst the families with a higher number of species, although the latter is slightly over-represented in Italy. Poaceae occur frequently in very stressful environments (Baskin and Baskin 2000) and, consequently, it is easy to understand why there is a high number of them, which can be considered as peculiar to gypsum substrates. Other conspicuous families in the taxonomic spectrum of the Italian gypsophytes are Apiaceae, Asteraceae, Plumbaginaceae and Crassulaceae. All of them are found in arid Mediterranean environments and, therefore, it is not surprising to find them so well represented in Italy.

Although there were variations in the abundance percentages, the most common families with the greatest match with the grouping occurred for the Spanish gypsophilous flora (Mota et al. 2011), where Asteraceae (14.1%), Brassicaceae and Plumbaginaceae (12.68%) correspond to the larger families. Nevertheless, Poaceae and Apiaceae obtained greater representation in the Italian gypsophilous flora. On the contrary, Lamiaceae, Fabaceae and Caryophyllaceae families appear to be less common on Italian gypsum outcrops with respect to other countries (Table 3).

Plant formations linked to gypsum substrates are usually dominated by small plant species such as chamaephytes or hemicryptophytes, similarly to those which occur in other Mediterranean areas, although there are exceptions of woody plant formations growing on gypsum (Pérez-García et al. 2017). The intense exposure to sunshine for these environments, the shortage of water and nutrient imbalance are probably responsible for this scenery. In addition, the abundance of therophytes may be explained by water shortage. Thus, some annual plants can be favoured on gypsum by drought (Merlo et al. 2001).

## Conclusions

Efforts to ensure the conservation of Mediterranean gypsophilous vegetation, considered as a Priority Habitat, should be focused on endangered, rare or endemic species, according to the premises established by the EU. All these efforts cannot be easily undertaken unless it is previously determined which species, out of many hundreds, are to be given top priority. The approach adopted in this work may help both to focus on certain species and to detect research and conservation priorities. The high proportion of Italian endemic species and the geographic rarity component of the flora associated with Italian gypsum outcrops is an aspect that makes these outcrops very interesting habitats. However, the degree of threat to the gypsophilous flora in Italy could have been insufficiently assessed. This fact is confirmed since less than 7% of the gypsophytes of the Italian Checklist have been evaluated under IUCN protocol. As there has been a prior effort to establish a network of sites for the conservation of nature (Natura 2000 Network), it would be worthwhile to extend this initiative to areas supporting Italian gypsum outcrops that have biodiversity values worthy of consideration. In order to achieve this purpose, an in-depth review of the conservation status for both the gypsophilous flora and the natural areas where these substrata occur in Italy is crucial. Data generated by experts in conservation, for which threat categories of red-listed species are based, should be incorporated into nature protection Acts (Mendoza-Fernández and Mota 2016) to ensure the preservation of these sites in Italy.

There are numerous SCI and SCZ including gypsum outcrops and their associated flora. The question that remains to be clarified is whether they are sufficient to ensure the conservation of this flora as well as the vegetation linked to this peculiar substrate. In this examination of gaps in conservation, fauna and other plant groups, such as lichens and bryophytes, should be integrated (Mota et al. 2011).

At this moment, the existence of endemic and rare flora with remarkable eco-morphological adaptations and the description of new taxa growing on gypsum outcrops fully justifies the conservation of these outcrops (Gallo 2014). Some Italian gypsum outcrops are currently under protection, but other areas have not yet been included in the network of nature reserves. As Panuccio et al. (2017) and Spampinato et al. (2018) pointed out for Calabria (South-Italy) and Mendoza-Fernández et al. (2014) for Andalusia (southern Spain), arid or semi-arid territories are often under-represented in protected area networks. This is the case for most Italian gypsum sites. Furthermore, the checklist of Italian gypsophytes should contribute to a better understanding of the autoecology and synecology of rare and endemic species and, therefore, to better conservation of the biodiversity associated with gypsum areas in Italy. For example, scrub communities, typical of Italian gypsum substrates, represent one of the major gaps in conservation habitats in the European Union. In order to fill this gap, the peculiar Italian gypsum habitats could be considered by modifying the meaning that habitat 1520* currently has in the manual of habitat interpretation and through the addition of Italian gypsophytes in order to include the gypsum habitat in Italy, amongst those of European interest.

Data provided in this paper denote an important advance in this sense, because only five plant species in Italy have been recognised as characteristic taxa for this habitat on the European Red List of Habitats (Loidi 2016). These are: Brassica villosa subsp. tineoi, *Chaenorhinum rupestre*, *Festuca gypsophila* (*Ctenopsis gypsophila*), *Erysimum metlesicsii* and Sedum gypsicola subsp. trinacriae. However, according to the information provided in this paper, at least twelve species, more than double, could be indicative plants for this Priority Habitat. Thus, the following should be added to those already mentioned: Petrosedum ochroleucum subsp. mediterraneum, *Allosorus persicus*, *Artemisia pedemontana*, Diplotaxis harra subsp. crassifolia and Stipa austroitalica subsp. frentana. In addition, some species typically related to saline soils belonging to the “Serie gessoso-solfifera” of Sicily, such as *Reaumuria vermiculata*, *Limonium catanzaroi* and *Limonium optimae*, may also occur.

## Appendix 1

**References appendix**

Aleffi, M., Pellis, G. & Puglisi, M. (2014). The bryophyte flora of six gypsum outcrops in the Northern Apennines Nature 2000 Network, Emilia Romagna Region, Italy. *Plant Biosystems*, 1484, 825–836.

Antolini, P. (1984). Rassegna dei principali affioramenti di gesso in Italia. *Atti della Accademia roveretana degli Agiati*, 24, 83–117.

Bazan, G., Ilardi, V., Minissale, P. & Sciandrello, S. (2006). La biodiversità vegetale di Monte Gibliscemi Mazzarino Sicilia. *Quaderni di Botanica ambientale e applicata*, 172, 121–140.

Biondi, E., Blasi, C., Allegrezza, M., Anzellotti, I., Azzella, M.M., Carli, E. et al. (2014). Plant communities of Italy: The Vegetation Prodrome. *Plant Biosystems*, 1484, 728–814.

Brullo, S., Marcenò, C., Minissale, P. & Spampinato, G. (1989). Su una nuova associazione del *Sedo-Ctenopsion gypsophilae* rinvenuta in Sicilia. *Archivio Botanico e Biogeografico Italiano*, 651(2), 100–108.

Cobau, R. (1932). Su la flora dei “gessi” bolognesi. *Nuovo Giornale Botanico Italiano*, 392, 313–345.

Conti, F. (1998). An annoted checklist of the flora of the Abruzzo. *Bocconea*, 10.

Conti, F. & Pirone, G. (1988). Segnalazioni Floristiche Italiane. *Informatore Botanico Italiano*, 20, 654–656.

Corbetta, F. (1964). Alcuni aspetti della vegetazione dei gessi Bolognesi. *Natura e Montagna*, 24, 30–37.

Di Falco, G., Manzi, A. & Manzi, G. (2003). *I gessi di Gessopalena e della valle dell’Aventino.* Un museo nel territorio. Editrice Ianieri, Pescara.

Di Martino, A., Marcenò, C. & Raimondo, F.M. (1976). Nota preliminare sulla vegetazione gipsofila della Sicilia centro-meridionale. *Giornale Botanico Italiano*, 111, 369–370.

Ferrari, C. (1974). La vegetazione delle rupi gessose di Miserazzano e della Croara, Bologna. *Notes de Fitosociologia*, 8, 65–74.

Ferro, G., Coniglione, P. & Oliveri, S. (1979). I praticelli effimeri su gesso nel territorio di Caltanissetta, Sicilia. *Atti dell’ Accademia Gioenia di Scienze Naturali in Catania*, 64, 137–141.

Gallo, L. (2014). *Sedum ochroleucum* subsp. *mediterraneumCrassulaceae*, a new Italian endemic. *Willdenowia*, 44, 27–33.

Gianguzzi, L., D’Amico, A., Caldarella, O. & Romano, S. (2010). Note distributive ed ecologiche su alcune rare entità della flora vascolare siciliana. *Il Naturalista Siciliano*, 342, 227–244.

Gianguzzi, L., D’Amico, A., Caldarella, O. & Romano, S. (2011). La flora vascolare delle Rocche di Entella entroterra della Sicilia occidentale. *Il Naturalista Siciliano*, 353(4), 363–405.

Giardina, G., Raimondo, F.M. & Spadaro, V. (2007). A catalogue of plants growing in Sicily. *Bocconea*, 20, 5–582.

Giusso del Galdo, G., Marcenò, C., Musarella, C.M. & Sciandrello, S. (2008). La vegetazione costiera R.N.O. ‘Torre Salsa’ Siculiana-AG. *Informatore Botanico Italiano*, 401, 73–89.

Macchiati, L. (1888). Contribuzione alla flora del gesso. *Nuovo Giornale Botanico Italiano*, 20, 418–422.

Macchiati, L. (1891). Seconda contribuzione alla flora del gesso. *Nuovo Giornale Botanico Italiano*, 23, 171–175.

Macchiati, L. (1892). Terza contribuzione alla flora del gesso. *Bullettino della Società Botanica Italiana*, 120–122.

Manzi, A. (1993). Note floristiche per le regioni Abruzzo e Marche. *Archivio Botanico*, 68, 13–180.

Marcenò, C., Falci, A. & Pasta, S. (2011). Su alcuni lembi di vegetazione pre-forestale e forestale della provincia di Enna Sicilia centrale. *Naturalista Siciliano*, 352, 295–312.

Marcenò, C. & Gristina, A.S. (2010). Su *Chaenorhinum rubrifolium* Dc. Fourr. *Scrophulariaceae*, specie nuova per la flora siciliana e sull’ecologia e distribuzione del genere *Chaenorhinum* DC. Reichenb. in Sicilia. *Naturalista Siciliano*, 343(4), 477–485.

Montanari, S., Bagli, L., Sirotti, M., Faggi, G. & Alessandrini, A. (2016). Flora dei gessi e solfi della Romagna Orientale. *Memorie dell’Instituto Italiano di Speleologia*, 31, 181–219.

Pasquini, D. (1944). La vegetazione dei gessi reggiani. *Atti della Società dei naturalisti e matematici di Modena*, 75, 264–282.

Pasta, S. (2001). Lineamenti della flora e della vegetazione del Lago Sfondato. *Naturalista Siciliano*, 4, 401–421.

Pasta, S. & La Mantia, T. (2001). Lineamenti della flora e della vegetazione dell’area della Riserva Naturale “Grotta di Santa Ninfa”. *Naturalista Siciliano*, 4, 271–297.

Privitera, M. (1989). La vegetazione muscinale dei gessi dell’Agrigentino, Sicilia occidentale. *Bollettino della Accademia Gioenia di Scienze Naturali*, 22, 105–113.

Raimondo, F.M., Domina, G. & Spadaro, V. (2010). Checklist of the vascular flora of Sicily. *Quaderni di Botanica Ambientale e Applicata*, 21, 189–252.

Troia, A., Pasta, S., Campo, G. & Romano, S. (1998). Indagini tassonomiche e corologiche sul genere *Sedum* L. serie Rupestria Berger Crassulaceae in Sicilia. *Naturalista Siciliano*, 4(221-2), 73–85.

Troia, A. (2002). *La flora gipsicola. Aspetti biologici ed ecologici delle piante che vivono sul gesso*. Palermo: Assessorato Territorio e Ambiente Regione Siciliana & Club Alpino Italiano.

## Supplementary materials

Supplementary material 1 Plant species data This is an open access article distributed under the terms of the Creative Commons Attribution License (CC BY 4.0), which permits unrestricted use, distribution, and reproduction in any medium, provided the original author and source are credited.Carmelo Maria Musarella, Antonio Jesús Mendoza-Fernández, Juan Francisco Mota, Alessandro Alessandrini, Gianluigi Bacchetta, Salvatore Brullo, Orazio Caldarella, Giampiero Ciaschetti, Fabio Conti, Luciano Di Martino, Amedeo Falci, Lorenzo Gianguzzi, Riccardo Guarino, Aurelio Manzi, Pietro Minissale, Sergio Montanari, Salvatore Pasta, Lorenzo Peruzzi, Lina Podda, Saverio Sciandrello, Leonardo Scuderi, Angelo Troia, Giovanni Spampinato

Supplementary material 2 ANOVA analysis by gypsophily level This is an open access article distributed under the terms of the Creative Commons Attribution License (CC BY 4.0), which permits unrestricted use, distribution, and reproduction in any medium, provided the original author and source are credited.Carmelo Maria Musarella, Antonio Jesús Mendoza-Fernández, Juan Francisco Mota, Alessandro Alessandrini, Gianluigi Bacchetta, Salvatore Brullo, Orazio Caldarella, Giampiero Ciaschetti, Fabio Conti, Luciano Di Martino, Amedeo Falci, Lorenzo Gianguzzi, Riccardo Guarino, Aurelio Manzi, Pietro Minissale, Sergio Montanari, Salvatore Pasta, Lorenzo Peruzzi, Lina Podda, Saverio Sciandrello, Leonardo Scuderi, Angelo Troia, Giovanni Spampinato
